# Long-term exposure to particulate matter was associated with increased dementia risk using both traditional approaches and novel machine learning methods

**DOI:** 10.1038/s41598-022-22100-8

**Published:** 2022-10-12

**Authors:** Yuan-Horng Yan, Ting-Bin Chen, Chun-Pai Yang, I-Ju Tsai, Hwa-Lung Yu, Yuh-Shen Wu, Winn-Jung Huang, Shih-Ting Tseng, Tzu-Yu Peng, Elizabeth P. Chou

**Affiliations:** 1 Department of Endocrinology and Metabolism, Kuang Tien General Hospital, Taichung, Taiwan; 2 Department of Medical Research, Kuang Tien General Hospital, Taichung, Taiwan; 3 Institute of Biomedical Nutrition, Hungkuang University, Taichung, Taiwan; 4 Department of Neurology, Neurological Institute, Taichung Veterans General Hospital, Taichung, Taiwan; 5 Department of Applied Cosmetology, Hungkuang University, Taichung, Taiwan; 6 Department of Neurology, Kuang Tien General Hospital, Taichung, Taiwan; 7 Department of Bioenvironmental Systems Engineering, National Taiwan University, Taipei, Taiwan; 8 Department of Safety, Health, and Environmental Engineering, Hungkuang University, Taichung, Taiwan; 9 Division of Endocrinology and Metabolism, Department of Internal Medicine, Kuang Tien General Hospital, Taichung, Taiwan; 10 Jenteh Junior College of Medicine, Nursing and Management, Miaoli County, Taiwan; 11 Department of Statistics, National Chengchi University, No. 64, Sec. 2, Zhinan Rd., Wenshan Dist., Taipei City, 116 Taiwan

**Keywords:** Risk factors, Environmental chemistry, Environmental impact

## Abstract

Air pollution exposure has been linked to various diseases, including dementia. However, a novel method for investigating the associations between air pollution exposure and disease is lacking. The objective of this study was to investigate whether long-term exposure to ambient particulate air pollution increases dementia risk using both the traditional Cox model approach and a novel machine learning (ML) with random forest (RF) method. We used health data from a national population-based cohort in Taiwan from 2000 to 2017. We collected the following ambient air pollution data from the Taiwan Environmental Protection Administration (EPA): fine particulate matter (PM_2.5_) and gaseous pollutants, including sulfur dioxide (SO_2_), carbon monoxide (CO), ozone (O_3_), nitrogen oxide (NO_x_), nitric oxide (NO), and nitrogen dioxide (NO_2_). Spatiotemporal-estimated air quality data calculated based on a geostatistical approach, namely, the Bayesian maximum entropy method, were collected. Each subject's residential county and township were reviewed monthly and linked to air quality data based on the corresponding township and month of the year for each subject. The Cox model approach and the ML with RF method were used. Increasing the concentration of PM_2.5_ by one interquartile range (IQR) increased the risk of dementia by approximately 5% (HR = 1.05 with 95% CI = 1.04–1.05). The comparison of the performance of the extended Cox model approach with the RF method showed that the prediction accuracy was approximately 0.7 by the RF method, but the AUC was lower than that of the Cox model approach. This national cohort study over an 18-year period provides supporting evidence that long-term particulate air pollution exposure is associated with increased dementia risk in Taiwan. The ML with RF method appears to be an acceptable approach for exploring associations between air pollutant exposure and disease.

## Introduction

According to the World Hospital Organization (WHO) in 2016^1^, approximately 92% of the world’s population lives in areas where air pollution is severe; furthermore, air pollution results in approximately 11.6% of deaths in the world (90% of these deaths occur in low- and middle-income countries). Air pollution exposure and adverse health effects are related to general mild physical illness, respiratory and cardiovascular diseases, and even cancer, respiratory and cardiovascular diseases that lead to death. Moreover, a wide range of acute and chronic health effects are aggravated by air pollution exposure. Experts firmly believe that air pollution is responsible for more deaths globally than acquired immunodeficiency syndrome (AIDS), malaria, and tuberculosis^2–4^. In addition to the effect on health, the recent unemployment rate is highly correlated with air pollutant levels. For instance, the 2008–2014 economic crisis has been shown to have influenced the air pollutant level^5^, and students’ academic performance has been found to be subject to air pollution levels^6^. Thus, air pollution is a current crisis and an issue of global concern and poses a particular risk to public health.

Previous epidemiological studies have suggested an association between air pollution exposure and the risk of dementia^7^. However, recent longitudinal studies have shown inconsistent associations between long-term exposure to ambient fine particulate matter (PM_2.5_), nitrogen dioxide (NO_2_), sulfate dioxide (SO_2_), and ozone (O_3_) and the incidences of dementia and Alzheimer's disease^8, 9^. Study design, air pollutants assessed, and statistical methods used may influence the outcomes. A continued need to confront methodological challenges in this line of research has been noted^10^. In addition, evidence of long-term air pollution exposure and the risk of dementia in East Asia cities is limited^11–14^. As East Asia is one of the worst air pollution regions globally, more research is needed.

In the past, environmental epidemiological studies primarily used traditional regression models to infer the relationship between environmental factors, such as temperature, humidity, and air pollutant levels, and disease. On the other hand, diseases have often only been controlled as interference factors in tested models, or a mere t test or ANOVA test has been used to illustrate significant differences^15–17^. Machine learning (ML) is a form of artificial intelligence that allows systems to learn from data. It has a wide range of applications and can also be used for nonlinear data without too many assumptions about the distribution of population data^18–20^. Ideally, the real purpose of data analysis is to bring out the visible message content contained in the data, which data analysts provide to interested decision-makers. This data-driven intelligence contributes to a deep understanding and knowledge of the data and provides the perfect basis for decision-makers. However, research using statistical and ML methods mainly focuses on the prediction of air pollutant concentrations^21, 22^, the relationship between air pollutant concentration environmental exposure and clinical data^23^, or the use of unsupervised learning methods to study regions based on air pollution indicators^24, 25^.

Recently, ML methods have been widely used in classification. These supervised learning methods are more stable and robust than the traditional model-based approaches. Supervised learning methods can address the curse of dimensionality and noise of the data and provide reliable prediction results via a speed computing process. Therefore, ML methods are increasingly used to generate predictions based on epidemiology datasets. There has been great interest in comparing model performance among different ML methods. Studies have found that ML methods such as logistic regression (LR), random forest (RF), support vector machine (SVM), gradient boosting machine (GBM), K nearest neighbor (KNN), and neural networks can improve clinical risk prediction and the identification of risk factors^26–30^. Weng et al.^31^ showed the value of RF and deep learning methods in traditional epidemiological studies. Chun et al.^32^ demonstrated the superior predictive value of ML methods compared with traditional Cox models. Moncada-Torres et al.^33^ showed that ML-based models can perform at least as well as classical Cox proportional hazard regression. Studies suggest that the RF method can be an alternative choice to Cox regression^34^. However, some studies have shown that ML methods such as RF, SVM, or artificial neural networks do not always perform better than Cox regression^35, 36^.

Due to the popularity of ML methods, more studies have used ML techniques for dementia prediction^37–39^. The RF method is one of the popular and preferable methods used by researchers. It has proven to be more effective in dementia prediction^40–44^ than other ML methods and is not hindered by the “black box” performance of certain ML approaches. The RF method can not only be used to predict results but also to show the importance of features used in prediction. In this study, we compared the performance of an ML method and traditional statistical survival models. We used clinical datasets to show the potential of ML methods compared to traditional Cox regression in predicting dementia risk based on air pollution exposure. We integrated the longitudinal data of 457,064 people in the National Health Insurance Research Database of Taiwan from 2000 to 2017 with monthly spatiotemporal-estimated air quality data.

Since air pollution exposure has been linked to dementia risk with inconsistent results and a novel method to explore the associations is lacking, the objective of this study was to investigate whether long-term exposure to ambient particulate air pollution, controlling for gaseous pollutants, increases dementia risk. Both the traditional Cox model and a novel ML with RF method were used.

## Materials and methods

### Medical records

We obtained a longitudinal registry of beneficiaries and medical records of outpatient and inpatient visits of two million people randomly selected from all insured beneficiaries from the National Health Insurance Database (NHIRD) from 2000 to 2017. The National Health Insurance (NHI) Program in Taiwan has a high coverage rate of 99.99%. The Health and Welfare Data Center (HWDC) of Taiwan’s Ministry of Health and Welfare (MOHW) continues to maintain the NHIRD and permits applications for data usage for research purposes. Details of the NHIRD are described elsewhere (Hsieh et al., 2019). All datasets are linked through unique encrypted personal identifiers. The disease diagnoses were defined by the International Classification of Diseases, 9th Revision, Clinical Modification (ICD-9-CM) before 2016 and the International Classification of Diseases, 10th Revision, Clinical Modification (ICD-10-CM) since 2016. The study was exempted from ethics review because deidentified data were utilized (Kuang Tien General Hospital Institutional Review Board approval document KTGH 10923), and the study was conducted according to the guidelines and regulations of the Declaration of Helsinki^45^. The NHIRD is a deidentified dataset for research purposes, so the requirement for informed consent was waived by the Ethics Research Committee of Kuang Tien General Hospital.

This study was approved by HungKuang University and Kuang Tien General Hospital (HK-KTOH-109-05). The Institutional Review Board of Kuang Tien General Hospital reviewed the protocol of the current study and waived the need for informed consent in view of the retrospective design (Approval IRB number: KTGH 10923) in 2020. All methods were carried out in accordance with relevant guidelines and regulations.

### Air quality and meteorological data

Data on hourly air quality variables, including temperature (°C), relative humidity (%), concentrations of sulfur dioxide (SO_2_; ppb), carbon monoxide (CO; ppm), ozone (O_3_; ppm), nitrogen oxide (NO_x_; ppb), nitric oxide (NO; ppb), nitrogen dioxide (NO_2_; ppb), particulate matter (PM_10_; μg/m3) and PM_2.5_ (μg/m3) from 2005 to 2017, were downloaded from the Taiwan Environmental Protection Administration (EPA) website (https://airtw.epa.gov.tw/CHT/Query/His_Data.aspx)^46^. There are 83 EPA air monitoring stations in Taiwan. The spatial distribution of monitoring stations was shown in our previous study^47^.

Daily average values of temperature and relative humidity were aggregated from hourly data. If the number of data points was under 75% on that day (less than 18 observations), the daily data were considered missing. There are no EPA air monitoring stations on Taiwan’s offshore islands, and residents of the offshore islands were excluded from this study.

This study applied a geostatistical approach, namely, the Bayesian maximum entropy method, to calculate the spatiotemporal estimation of hourly ambient concentrations for each township in Taiwan from 2005 to 2017^47^. We converted spatiotemporal-estimated hourly data to daily average data and monthly average data.

The dataset of the registry of beneficiaries contains monthly records of the demographic data for each insurer, including birth year, sex, residential county and township, and insured status. Therefore, each subject's residential county and township were reviewed monthly and linked to air quality data based on the corresponding township and month of the year for each subject.

### Study subjects

A total of 469,081 insurers aged above 50 years on January 1, 2005, were included in our study. We excluded 11,500 subjects with a previous dementia diagnosis (ICD9 CM codes: 290, 294.1, 331.2; ICD10 CM codes: F00, F01, F02, F03, F05.1, G30, G31.1) and 517 subjects living on the offshore islands due to missing air quality data. Finally, we included 457,064 subjects in our study. The study index date was January 1st, 2005. All subjects were followed from the index date to the occurrence of dementia, termination of insurance, or December 31, 2017, whichever came first.

Since air quality varies with time and the CCI score may also vary with time, we created yearly records consisting of baseline characteristics, annual concentration of air pollutants per interquartile range (IQR), annual temperature, and annual relative humidity for each subject from the index date to the end of follow-up. The outcome status was recorded every year. If a subject had no dementia at the end of follow-up, then the outcome status was censored for every yearly record for the subject. Otherwise, the outcome status was recorded as an event in the last year for the subject. There were 13 yearly records at most for a subject.

### Comorbidities

Comorbidities including hypertension (HTN; ICD9 CM codes: 401–405; ICD10 CM codes: I10-I15), diabetes (DM; ICD9 CM code: 250; ICD10 CM codes: E10-E14), hyperlipidemia (HL; ICD9 CM code: 272; ICD10 CM code: E78), and the Charlson Comorbidity Index (CCI score)^48, 49^ before the index date were considered potential risk factors for dementia. We used hospital admission records to calculate the modified CCI score at baseline and during the follow-up period, which excluded dementia since dementia was the primary outcome in this study.

### Statistical analysis

To compare the baseline characteristics between people with and without dementia diagnosis at the end of follow-up, we used the chi-square test for categorical variables and the Wilcoxon rank-sum test for continuous variables.

To avoid collinearity, we calculated Pearson correlation coefficients for all two-variable combinations of temperature, relative humidity, and air pollutants. An absolute value of Pearson’s correlation coefficients > 0.7 was considered highly correlated, and we only selected variables that had lower correlations with each other in the further analysis.

We examined multiple pollutants simultaneously in our analyses to study the effect of particulate air pollution (PM_2.5_) in a single-pollutant model, two-pollutant model, and three-pollutant model to assess the association between PM_2.5_ and dementia.

We performed extended Cox models to analyze the association between ambient particulate matter and the risk of developing dementia. Since we have time-varying variables in our model, the Cox regression model based on the Andersen-Gill counting process was used for analysis. We used time-dependent ROC curve estimation with the R packages survivalROC^50^ and rms^51^ to measure its performance by the concordance index (C-index)^52^.

The RF^53^ approach is a popular supervised method due to its computational efficiency and nonoverfitting characteristic. It is an ensemble method that is used to construct multiple decision trees. The trees are built using a bagging approach to sample a subset of the training data and randomly select features for the learning process. Prediction is made by aggregating the predictions of the ensemble. The RF method can be used to rank the importance of features that can discriminate the target feature. It has been successfully applied to various practical problems due to the accuracy of its performance. Air quality data for the RF method were aggregated from yearly records to determine the 1-, 3-, 5-, and 10-year averages. The CCI score during the study period was chosen as the last observation of the yearly record. The selected features used to predict dementia status were age, sex, modified CCI, and baseline comorbidities, including HTN, DM, HL, temperature, relative humidity, PM_2.5_, CO, SO_2_, NO, NO_2_, NO_x_, and O_3_. The sensitivity and specificity of the predicted results were calculated to generate a receiver operating characteristic (ROC) curve. The area under the ROC curve (AUC) for each dataset is reported to compare the accuracy of the two models. The computation of RF was carried out by using the R package randomForest^54^, and AUCs were computed by using the R package pROC^55^. We used 1000 trees in this study.

All statistical analyses were performed using R software (R Core Team, 2021; https://www.R-project.org/) and SAS software, Version 9.4 (SAS Institute Inc., Carey, NC, USA).

## Results

There were 457,064 participants in this study. The mean age of the participants was 63 ± 9.9 years, the proportion of males and females was approximately 1:1, and the mean follow-up period was 10.8 ± 3.7 years. Baseline comorbidities are shown in Table 1. We also compared the characteristics of the participants with and without a dementia diagnosis at the end of follow-up. The mean age of the participants with dementia was 10 years older than that of the participants without dementia (71.4 vs. 61.8 years, respectively). The proportion of females among the participants with dementia was higher than that among those without dementia (54.4% vs. 49.6%, respectively). Baseline comorbidities were more prevalent in the participants with dementia than in those without dementia.

**Table 1:** Baseline characteristics, n (%).

	All participants	Dementia events over the 13-year follow-up		*P* value
		Nonevents	Events	
	(n = 457,064)	(n = 400,032)	(n = 57,032)	
**Age at baseline, years**				
Mean (SD)	63 (9.9)	61.8 (9.4)	71.4 (8.7)	< 0.0001
Sex				
Men	227,448 (49.8)	201,437 (50.4)	26,011 (45.6)	< 0.0001
Women	229,616 (50.2)	198,595 (49.6)	31,021 (54.4)	
**Duration of follow-up, years**				
Mean (SD)	10.8 (3.7)	11.4 (3.4)	7.1 (3.7)	< 0.0001
Baseline comorbidity				
Hypertension	207,256 (45.3)	170,740 (42.7)	36,516 (64.0)	< 0.0001
Diabetes	95,129 (20.8)	78,329 (19.6)	16,800 (29.5)	
Hyperlipidemia	121,988 (26.7)	103,123 (25.8)	18,865 (33.1)	
Modified CCI*				
0	372,606 (81.5)	331,622 (82.9)	40,984 (71.9)	< 0.0001
1	25,143 (5.5)	20,650 (5.2)	4493 (7.9)	
2	13,475 (2.9)	11,217 (2.8)	2258 (4.0)	
3	4882 (1.1)	4019 (1.0)	863 (1.5)	
≥ 4	40,958 (9.0)	32,524 (8.1)	8434 (14.8)	
Number of townships	338			

*Modified CCI, which excluded dementia.

**Figure 1:**
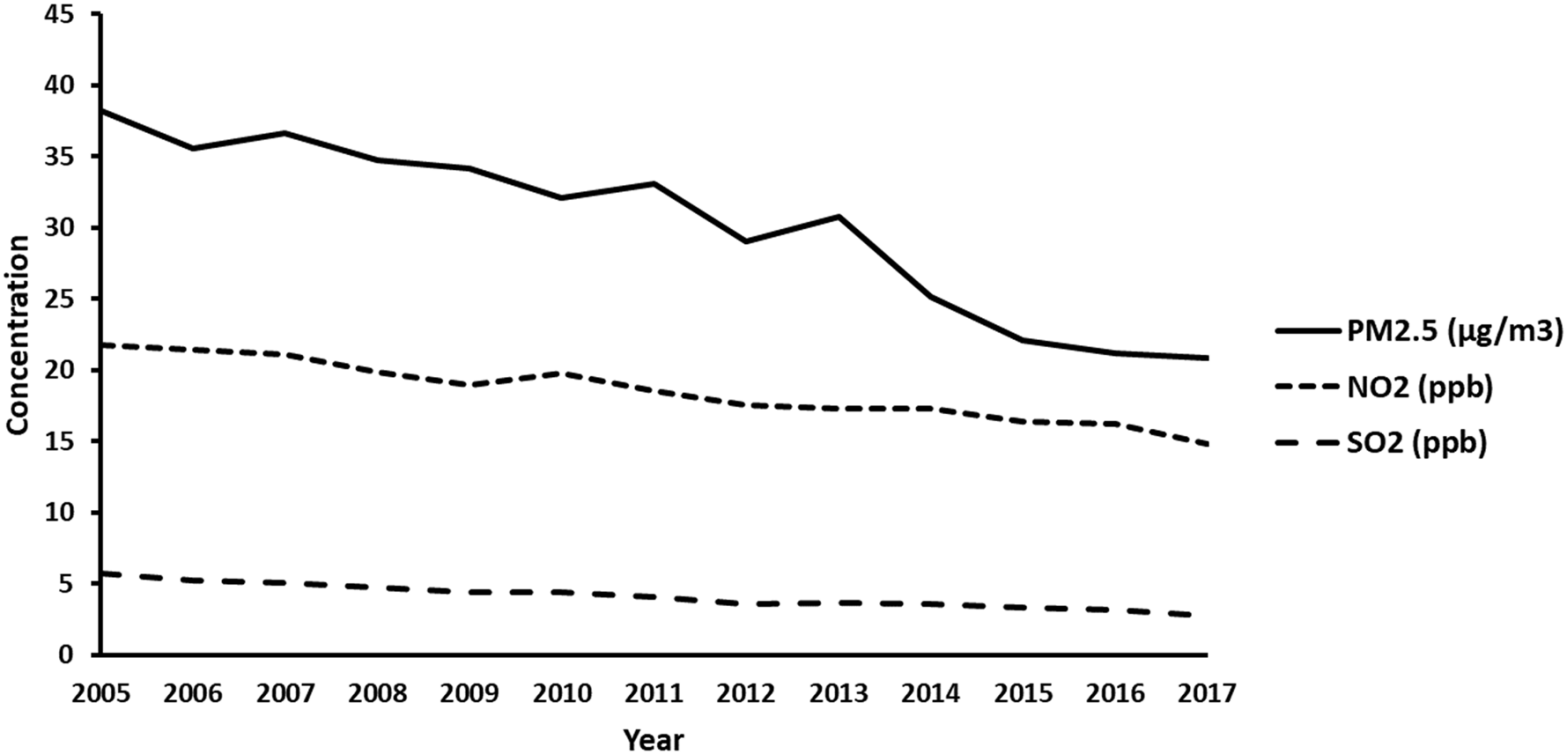
shows the temporal distribution of PM_2.5_, NO_2_, and SO_2_ used in this study.

Figure 1 shows the change in concentrations of air pollutants over time. The concentrations of PM_2.5_, NO_2_, and SO_2_ decreased. The participants’ mean exposure levels to air pollutants during the follow-up period are shown in Table 2.

**Table 2:** Participants’ mean exposure levels to air pollutants during the follow-up period.

Air pollutant	Mean (SD)	Median	Range	IQR
PM_2.5_	31.76(6.73)	31.49	10.42–72.66	10.29
PM_10_	56.31(11.37)	54.34	24.26–115.66	19.22
CO	0.57(0.16)	0.53	0.1–1.69	0.21
NO	8.84(5.84)	6.59	0.1–52.61	7.11
NO_2_	18.97(4.57)	18.38	2.83–44.25	7.23
NOx	27.63(9.98)	25.18	4.71–93.61	13.46
O_3_	27.53(2.3)	27.7	12.15–44.31	3.35
SO_2_	4.35(1.35)	3.92	1.39–20.82	1.33

IQR = the 75th percentile–the 25th percentile.

Table 3 shows the association between PM_2.5_ per IQR and the risk of dementia using a single-pollutant model, two-pollutant model, and three-pollutant model. Increasing the concentration of PM_2.5_ by one IQR increased the risk of dementia by approximately 5% in the single-pollutant model (HR = 1.05 with 95% CI = 1.04–1.05). In the two-pollutant model, increasing the concentration of PM_2.5_ by one IQR increased the risk of dementia by approximately 11% when considering SO_2_ in the model (HR = 1.11 with 95% CI = 1.10–1.12). Increasing the concentration of PM_2.5_ by one IQR increased the risk of dementia by approximately 3% when considering NO_2_ in the model (HR = 1.03 with 95% CI = 1.03–1.04). In the three-pollutant model, increasing the concentration of PM_2.5_ by one IQR increased the risk of dementia by approximately 10% when considering NO_2_ and SO_2_ in the model (HR = 1.10 with 95% CI = 1.09–1.11).

**Table 3:** Hazard ratios (95% CI) for the association between PM_2.5_ and dementia risk during the 13-year follow-up period. Adjusted for age, sex, modified CCI, hypertension, diabetes, hyperlipidemia, temperature, relative humidity and pollutants in the corresponding year.

	Hazard ratio* (95% CI)
PM_2.5_	1.05 (1.04, 1.05)
PM_2.5_ + SO_2_	1.11 (1.10, 1.12)
PM_2.5_ + NO_2_	1.03 (1.03, 1.04)
PM_2.5_ + SO_2_ + NO_2_	1.10 (1.09, 1.11)

Table 4 shows the comparison of the performances of the extended Cox model with the RF approach. The prediction accuracy of the RF method was approximately 0.7, but the AUC was lower than that of the Cox model. The results clearly showed that discrimination is better with the Cox model. In addition, the AUC results at 1, 3, and 5 years were stable for both methods (0.79 for the Cox model and 0.76 for the RF model) and only decreased by 1% as the prediction time increased to 10 years. The prediction accuracy of the RF method was stable as the prediction time increased.

**Table 4:** Performance of the Cox model and the random forest classification model. Cox model adjusted for age, sex, modified CCI, hypertension, diabetes, hyperlipidemia, temperature, relative humidity, PM_2.5_, NO_2_, and SO_2_ in the corresponding year. Random forest classification: age, sex, modified CCI, hypertension, diabetes, hyperlipidemia, temperature, relative humidity, PM_2.5_, CO, SO_2_, NO, NO_2_, NO_x_, and O_3._

	Cox model	Random forest	
	AUC	AUC	Accuracy
Year 1	0.79	0.76	0.70
Years 1–3	0.79	0.76	0.70
Years 1–5	0.79	0.76	0.70
Years 1–10	0.78	0.75	0.69

## Discussion

This national cohort study over an 18-year period provides supporting evidence that long-term ambient PM_2.5_ is associated with incident dementia in Taiwan, East Asia^9, 56–58^. This study had a much higher, PM_2.5_ level (31.7), above US National Ambient Air Quality Standards, than other studies. The findings imply that dementia could be powerfully prevented in highly polluted regions by air pollution control policies. We further examined multiple pollutants simultaneously in our analyses. After controlling for gaseous pollutants, PM_2.5_ showed consistent significant associations with dementia risk. The combination of PM_2.5_ and SO_2_ seemed to have the largest effects on dementia risk. Further studies are ongoing to investigate the role of gaseous pollutants and the synergistic effects of PM_2.5_ and multiple gaseous pollutants.

Although the performance was not superior to that of the Cox model, the ML with RF method appears to be an acceptable approach to exploring associations between air pollutant exposure and disease. This raises a potential methodological advance for an unknown link in environmental epidemiology^59^. The ML method is used mainly for source apportionment, forecasting/prediction of air pollution/quality or exposure, and generating hypotheses regarding air pollution epidemiology^18, 60, 61^. Using high-quality health data from the NHIRD and air quality data from Taiwan EPA monitoring stations, ambient air pollution has been linked to a wide variety of diseases in Taiwan^62–66^. Most of these studies used the Cox proportional hazards model with a generalized equation to estimate the association between exposure to air pollutants and the incidence, progression, and mortality associated with certain diseases. Using the ML method, we broaden the possibilities for linkage of environmental data with information from health databases. More associations will be identified with the accumulation of NHIRD and EPA monitoring data. ML models for predicting the incidence of disease using environmental and air pollution factors could evolve into medical and public health warning systems^67^.

Human and toxicological studies have provided evidence that air pollution induces brain toxicity^68–70^. Increased oxidative stress, inflammation, mitochondrial dysfunction, microglial activation, disturbance of protein homeostasis, and ultimately neuronal death often postulate and concomitantly coincide with the main mechanisms of air pollution-related neurodegenerative processes^71^. Further investigations are needed to understand the biological impact of air pollution on various types of neurodegeneration.

In such an era of abundant data, these data resources hide anonymous information or undiscovered principles. It is necessary to mine valuable information from these data, extract naturally encoded knowledge and intelligence, and understand the black box behind AI. Once these relevant characteristic factors have been identified, scientists can fully support their decision-making with interpretable and visible patterns, thus taking responsibility for decision-making. For researchers in the healthcare industry, this will connect their diagnostic decisions to an intricate set of responsibilities and consequential legitimacy.

ML methods are hypothesis-free and can be applied to different kinds of data, such as nonlinear or nonnormal data. These methods can be easily applied without having prior knowledge of the data shape. ML methods are not like traditional statistical methods that require careful model assumptions of data normality and feature independence. Thus, ML methods are more attractive for analyzing real-world datasets. Traditional statistical approaches, such as Cox regression, are limited in the number of features that can be included in a single model. ML methods can handle high-dimensional data that the Cox model does not^72^. In the future, other ML or deep learning methods should be considered. Ensemble methods that combine traditional statistical methods and ML approaches should be considered in future studies. Feature selection with ML methods can be applied first to identify the relevant risk factors in dementia prediction. More relevant features should be considered in the model to make ML methods more accurate. In addition to the supervised method, unsupervised clustering methods can be used to group patients with similar characteristics. For different groups of patients, we can further explore diverse predictive risk features.

The strengths of this study include the large national cohort random sample, over an 18-year observation period, and the novel method used. There were, however, several limitations in this study. First, although we adjusted for potential confounders, unrecognized confounders may have affected the results. Data on risk factors for dementia, such as smoking, alcohol intake, diet and exercise, were not present in our claims database, which prevented us from further exploring the potential effects of these variables. Second, dementia is a neurodegenerative disease that has a long insidious onset, which might have started long before being diagnosed. In this study, we used a rigorous definition of claim-based diagnosis. This may have led to covariate measurement misclassification. Third, given that PM_2.5_ is a heterogeneous mixture from multiple sources, the results are not generalizable to areas with different pollutant constituents and particle sources. Fourth, exposure misclassification is a common concern in environmental epidemiology. We did not have individual exposure data, which may have resulted in differential measurement errors. However, using modeled pollution from temporally resolved daily pollutant outputs at a fine spatial resolution, rather than monitored pollution data, may provide a more accurate exposure–response relationship and thereby substantially reduce the likelihood of exposure misclassification. Fifth, the associations of PM_2.5_ exposure with dementia subtypes were not examined in this study.

## Conclusion

This national cohort study of data collected over an 18-year period provides supporting evidence that long-term particulate air pollution exposure is associated with increased dementia risk in Taiwan. The ML with RF method appears to be an acceptable approach to exploring associations between air pollutant exposure and disease. The results highlight the potential value of expanding the use of ML in environmental epidemiological practice.

## Data Availability

The datasets generated and/or analyzed during the current study are available from the National Health Insurance Database (NHIRD), which has been transferred to the Health and Welfare Data Science Center (HWDC) and is a publicly available dataset. Available from: https://www.nhi.gov.tw/English/Content_List.aspx?n=8FC0974BBFEFA56D&topn=ED4A30E51A609E49. Accessed June 15, 2022.
